# Studies in the Use of Magnetic Microspheres for Immunoaffinity Extraction of Paralytic Shellfish Poisoning Toxins from Shellfish

**DOI:** 10.3390/toxins3010001

**Published:** 2011-01-04

**Authors:** Raymond Devlin, Katrina Campbell, Kentaro Kawatsu, Christopher Elliott

**Affiliations:** 1 Institute of Agri-Food and Land Use, School of Biological Sciences, Queen’s University Belfast, Stranmillis Road, Belfast, BT9 5AG, Northern Ireland, UK; Email: rdevlin04@qub.ac.uk (R.D.); Katrina.Campbell@qub.ac.uk (K.C.); 2 Division of Bacteriology, Osaka Prefectural Institute of Public Health, Osaka, Japan; Email: kawatu@iph.pref.osaka.jp

**Keywords:** immunoaffinity, paralytic shellfish poisoning (PSP), magnetic microspheres, Ferrospheres, saxitoxin

## Abstract

Paralytic shellfish poisoning (PSP) is a potentially fatal human health condition caused by the consumption of shellfish containing high levels of PSP toxins. Toxin extraction from shellfish and from algal cultures for use as standards and analysis by alternative analytical monitoring methods to the mouse bioassay is extensive and laborious. This study investigated whether a selected MAb antibody could be coupled to a novel form of magnetic microsphere (hollow glass magnetic microspheres, brand name Ferrospheres-N) and whether these coated microspheres could be utilized in the extraction of low concentrations of the PSP toxin, STX, from potential extraction buffers and spiked mussel extracts. The feasibility of utilizing a mass of 25 mg of Ferrospheres-N, as a simple extraction procedure for STX from spiked sodium acetate buffer, spiked PBS buffer and spiked mussel extracts was determined. The effects of a range of toxin concentrations (20-300 ng/mL), incubation times and temperature on the capability of the immuno-capture of the STX from the spiked mussel extracts were investigated. Finally, the coated microspheres were tested to determine their efficiency at extracting PSP toxins from naturally contaminated mussel samples. Toxin recovery after each experiment was determined by HPLC analysis. This study on using a highly novel immunoaffinity based extraction procedure, using STX as a model, has indicated that it could be a convenient alternative to conventional extraction procedures used in toxin purification prior to sample analysis.

## 1. Introduction

**Figure 1:**
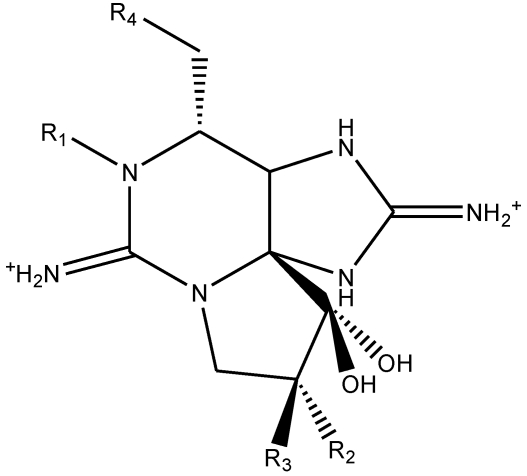
Structures of the PSP toxins.

Paralytic shellfish poisoning (PSP) is a potentially fatal human health condition which may occur after the consumption of PSP toxins in contaminated shellfish. The toxins are produced predominantly by dinoflagellates, in particular those from the *Alexandrium* species and are concentrated in shellfish which filter feed upon them. The neurotoxins responsible for PSP contain over 21 known analogues based upon the parent compound saxitoxin [1,2]. The toxins can be divided into two sub-groups based upon the presence or absence of a hydroxyl group at position R1. These two groups can be termed the saxitoxins (non-R1-hydroxylated toxins) and the neosaxitoxins (R1-hydroxylated toxins) [3] as shown in Figure 1 and Table 1.

The PSP toxins bind reversibly to voltage-gated sodium channels on excitable cell membranes and prevent channel opening. This leads to a reduction in the number of active sodium channels and a decrease or stop in the action potential of neurons or muscle cells. Various neurological symptoms which include perioral paraesthesia, dizziness, ataxia, dysphagia, diplopia and death by respiratory paralysis have been documented after consumption of PSP toxin contaminated shellfish [4,5].

In Europe the reference methods of monitoring are either the mouse bioassay (AOAC 959.08) [6] or the so called ‘Lawrence’ AOAC Official HPLC Method 2005.06 [7]. Both these methods, however, require the PSP toxins to be extracted via rather time consuming and complex methods. Sample extraction for any analytical method is a vital step in the process of producing a valid analytical result. There is substantial interest in developing selective extraction procedures for sample clean-up and/or extraction which eliminate matrix interferences before analysis or the preparation of reference standards. Solid phase extraction (SPE) has been used throughout the development of HPLC, particularly pre-column oxidation HPLC methods, as the standard clean-up method for PSP toxins [8,9,10,11]. However, the preparation of monoclonal antibody immunoaffinity columns for sample clean-up or analyte enrichment of PSP toxins before toxin analysis and quantification has been previously reported [12,13,14]. The reports concluded that due to the highly specific antibody-antigen interaction and removal of all matrix interferences (no sample matrix peaks present on the chromatograms) that immunoaffinity methods imply the potential to strongly improve the analysis of PSP toxins.

A further immuno-extraction procedure that may be used is the coupling of antibodies to magnetic microspheres. The microspheres used throughout the present study were a novel form of magnetic bead referred to as Ferrospheres-N. The -N signifies that their surface has been amine functionalized for the conjugation of antibodies and other ligands. They are buoyant, super-paramagnetic iron oxide coated hollow glass microspheres. On standing they will float to the surface and can be easily removed using a magnetic pen (Magpen) and then released into another solution and/or manipulated with a magnetic particle concentrator (MPC). This in turn allows thorough cleaning of the Ferrospheres-N and extends their reusability. These microspheres may have a possible application for the extraction or capture of toxins from complex matrices.

The standard extraction procedures require the use of either hydrochloric acid (mouse bioassay; [6]) or acetic acid (HPLC; [7]) for toxin extraction from homogenized shellfish samples. These two solutions both have low pH values and therefore may not be suitable for the immuno-capture of PSP toxins from shellfish extract using the GT-13A coupled Ferrospheres-N. Bates *et al.* (1978) [15] suggested that sodium acetate buffer (pH 5.0) may be successfully used for extraction of PSP toxins from shellfish tissue and thus was trialed in this study. Phosphate buffered saline (PBS) solution as an extraction buffer, was also tested in this study due to its physiological pH (pH7.2). The higher pH of these solutions may be more beneficial to immunoaffinity extraction of PSP toxins than the more traditionally used acidic solutions.

**Table 1:** Classification of major PSP toxins based on chemical structures.

			Carbamate Toxins	*N*-Sulfocarbamoyl toxins	Decarbamoyl toxins	Deoxydecarbamoyl toxins
R_1_	R_2_	R_3_	R_4_: OCONH_2_	R_4_: OCONHSO_3_^-^	R_4_: OH	R_4_: H
H	H	H	STX	B1 (GTX 5)	dc-STX	do-STX
H	H	OSO_3_^-^	GTX 2	C1	dc-GTX 2	do-GTX 2
H	OSO_3_^-^	H	GTX 3	C2	dc-GTX 3	do-GTX 3
OH	H	H	NEO	B2 (GTX 6)	dc-NEO	
OH	H	OSO_3_^-^	GTX 1	C3	dc-GTX 1	
OH	OSO_3_^-^	H	GTX 4	C4	dc-GTX 4	

The aim of the present study was to establish the practicability of coupling a monoclonal antibody to the magnetic Ferrospheres-N microspheres and to assess whether these microspheres could then be utilized in the extraction of low concentrations of STX not only from both spiked extraction buffers and spiked mussel extracts as model systems but also PSP toxins from naturally contaminated samples.

## 2. Materials and Methods

### 2.1. Chemicals and Reagents

Acetic acid, acetonitrile, adipic acid dihydrazide, ammonium acetate, ammonium formate (HPLC grade), disodium hydrogen phosphate, glycine, HPLC grade water, hydrochloric acid solution, hydrogen peroxide solution, orthophosphoric acid solution, periodic acid, potassium chloride, potassium di-hydrogen phosphate, sodium acetate, sodium carbonate, sodium cyanoborohydride, sodium hydroxide (NaOH) solution were purchased from Sigma-Aldrich (Dorset, UK). Glutaraldehyde solution (50%) was purchased from Polysciences (Eppelheim, Germany). All chemicals and solvents were of Analar grade or purer unless otherwise stated.

### 2.2. Materials

MAbTrap kit for monoclonal antibody purification was obtained from Amersham Biosciences (Amersham, Buckinghamshire, UK). Vivaspin centrifuge tubes were purchased from Sigma-Aldrich, Dorset, UK. Dialysis cassettes and a Bicinchoninic acid (BCA) protein assay kit were purchased from Pierce (Rockford, USA). Amine-coated hollow glass magnetic microspheres (Ferrospheres-N) and a Magpen were purchased from Microsphere Technology, Edinburgh, Scotland. The Ferrospheres-N are composed of a hollow glass core with a super-paramagnetic iron oxide surface, which is amine functionalized, giving a final diameter of ~20 µm. The Magpen is a metal pen-like tool containing a small, powerful magnet which resides in the lower shaft of the pen. Depressing the plunger moves the magnet into the tip of the pen and then it can be used to collect the buoyant Ferrospheres-N from the surface of the liquid before transfer to a fresh container. A Dynal magnetic particle concentrator (MPC) was purchased from Invitrogen (Paisley, Scotland).

### 2.3. PSP Toxin Standards

Saxitoxin dihydrochloride (STXdiH-65 μM), neosaxitoxin (NEO-65 μM), gonyautoxin 1/4 (GTX1-106 μM:GTX4-35 μM), gonyautoxin 2/3 (GTX2-118 μM:GTX3-39 μM), decarbamoyl saxitoxin (dcSTX-62 μM), decarbamoyl neosaxitoxin (dcNEO-30 μM), decarbamoyl gonyautoxin 2/3 (dcGTX2-114 μM:dcGTX3-32 μM), gonyautoxin 5 (GTX5-65 μM) and C1/C2 (C1-114 μM:C2-35 μM) as certified standard reference standard material were obtained from the Institute for Marine Biosciences, National Research Council, Halifax, Canada.

### 2.4. Mussel Samples

Batches of Blue mussel (*Mytilus edulis*) were harvested from Carlingford Lough, Ireland and analyzed by HPLC [7]. No detectable levels of PSP toxins were found.

Two contaminated mussel samples were collected from Ronas Voe, at the northwest coast of Northmavine in the north of the Shetland mainland and from Cribba Sound in the Shetland Islands. Both samples were confirmed to be contaminated with PSP toxins via HPLC analysis [7].

### 2.5. Production of Monoclonal Antibody (GT-13A)

A monoclonal antibody designated GT-13A raised to gonyautoxin (GTX) 2/3-keyhole limpet hemocyanin (KLH) protein conjugate was used. The production and characterisation of this MAb by ELISA and surface plasmon resonance have been described previously in detail elsewhere [16,17]. The GT-13A was produced in cell line and concentrated in cell culture media using Viva spin centrifuge tubes (30,000 MWCO) followed by purification via affinity chromatography using a protein G-sepharose gel column (MAbTrap Kit). Dialysis, over 24 hours in PBS buffer solution with three solution changes, was performed to remove any chemicals which may interfere with antibody coupling. The MAb was monitored by absorbance at 280 nm using a spectrophotometer.

### 2.6. Ferrospheres-N (Hollow Glass Magnetic Microspheres)

#### 2.6.1. Coupling of Monoclonal Antibody GT-13A to Ferrospheres-N

GT-13A was covalently coupled to the amine-coated Ferrospheres-N according to the manufacturer's instructions. Initially, three aliquots each containing 25 mg of Ferrospheres-N were incubated for 2 hours with 10% glutaraldehyde solution to allow sphere surface activation. Subsequently, 1.25 mg of GT-13A in PBS was added to the Ferrospheres-N for coupling by incubation at room temperature for 18 hours with constant agitation. A volume of 125 µL 1M sodium cyanoborohydride was added to the microsphere/PBS mixture and constantly agitated for 30 minutes, followed by addition of 225 µL of 100 mg/mL adipic dihydrazide to the mixture and a further incubation at room temperature with constant agitation for 30 minutes. The Ferrospheres-N were washed six times with PBS, three times with deionised water and finally washed three times each with 50 mM sodium acetate (pH 4.0, containing 300 mM NaCl), deionised water, 50 mM sodium carbonate (pH 9.0, containing 300 mM NaCl) and deionised water again, in that order. The Ferrospheres-N were then stored in PBS at 4 °C with 0.02% sodium azide, as a preservative, until use.

#### 2.6.2. Determination of Antibody Binding Capacity of the Ferrospheres-N

The Pierce BCA Protein Assay was used according to the manufacturer's instructions with some minor modifications. Between 5 and 6 mg of Ferrospheres-N were weighed into a 1.5 mL rmicrocentrifuge tube in triplicate. The Ferrospheres-N microspheres were then coupled to the GT-13A as described previously. After the removal of the final deionised water wash the Ferrospheres-N were reacted with the working reagent at 37 °C for 30 min, with inversion several times at 10 minute intervals. At the end of the incubation period the Ferrospheres-N were removed using the Magpen and the absorbance at 562 nm of each sample solution was measured. The values obtained were compared to a standard curve made from the bovine serum albumin (BSA) standards supplied and the mass of monoclonal antibody, GT-13A, per gram of microspheres was estimated.

#### 2.6.3. Elution Conditions

Dietrich *et al.*, 1998 [12] showed that glycine/HCl buffer (0.1 M, pH 2.5) allowed the quantitative elution of PSP toxins from coupled immunoaffinity columns without causing irreversible denaturation of the antibodies. Therefore, to determine if glycine/HCl buffer was suitable for elution of STX from GT-13A coupled Ferrospheres-N a series of investigations were carried out. In summary, a 1 mL volume of 100 ng/mL STX standard in PBS was added to a 25 mg batch of GT-13A coupled Ferrospheres-N, followed by a triplicate 1 mL wash of PBS and elution with 1 to 5 mL of glycine/HCl buffer.

#### 2.6.4. Ferrosphere-N Reusability

A batch of 25 mg of GT-13A coupled Ferrospheres-N was retested after a number of uses to test the performance of the Ferrospheres-N. The binding-elution described previously was repeated a number of times for various experiments and then after a set number of uses (n = 10), STX at a concentration of 100 ng/mL, in sodium acetate buffer, was added to the Ferrospheres-N, the microspheres were then washed with PBS, eluted with 0.1 M glycine/HCl buffer and cleaned with 4.0 M sodium chloride wash buffer before toxin determination by HPLC [7]. The microspheres were equilibrated with at least three 1 mL washes of PBS before and after use.

#### 2.6.5. STX Extraction from Spiked Extraction Buffers and Spiked Mussel Extracts

Sodium acetate (pH5) and PBS (pH7.2) extraction buffers were spiked with varying concentrations of saxitoxin in order to determine the toxin capture with the Ferrospheres-N in each buffering system. Triplicate 25 mg batches of microspheres were incubated with 1 mL of each of the STX spiked buffers at room temperature with constant agitation. The Ferrospheres-N microspheres were then washed three times with PBS to remove any unbound toxin. Bound toxin was eluted with the addition of 0.1 M glycine/HCl (pH 2.5) and agitation for 2 minutes.

Uncontaminated mussel samples from Carlingford Lough, Ireland were used to assess the applicability of the microspheres in mussel extracts to determine if this sample matrix affected the capture of STX from either of the extraction buffers. Briefly, uncontaminated mussel samples were removed from their shells, homogenized and the homogenate stored frozen at −20 °C until required. Samples (1 g of homogenate) were then weighed into centrifuge tubes and 4 mL of either sodium acetate buffer (pH5) or PBS buffer (pH 7.2) was added. Each tube was vortex mixed for 10 seconds. After mixing, samples were centrifuged at 3600*g* for 10 min at room temperature and the supernatants for each buffer were collected. Both sodium acetate and PBS uncontaminated mussel extracts were then spiked with STX at varying concentrations. The same procedure for the microspheres was used with spiked sodium acetate and PBS mussel extracts as with the buffers only. 

The effects of the STX concentration, incubation time and temperature on STX binding-elution from the mussel extracts were studied. For determination of the effect of initial concentration of STX on binding, the concentration added to the Ferrospheres-N ranged from 20-300 ng/mL, at room temperature with constant agitation for 10 minutes. To investigate the effect of time on STX recovery, mussel extracts were artificially contaminated with STX at a concentration of 100 ng/mL and a 1 mL aliquot of the extracts was added to 25 mg of GT-13A coupled Ferrospheres-N and incubated for a range of times (1-30 minutes). To determine whether or not temperature played an important role in the capture of STX, aliquots of the spiked extracts containing 100 ng/mL of STX were added to 25 mg of GT-13A coupled Ferrospheres-N. The extracts were incubated with the Ferrospheres-N for 10 minutes at a range of temperatures (4 °C, 10 °C, 22.5 [recorded room temperature], 37 °C). For each concentration, time and temperature experiment performed the binding-elution procedure was repeated three times. The STX in each stage was quantified using the AOAC Official Method 2005.06 [7]. Incubations at all time points tested were repeated in triplicate.

#### 2.6.6. PSP Toxin Extraction from Naturally Contaminated Mussel Samples

Contaminated mussel samples from the Shetlands were used to assess the applicability of the microspheres in naturally contaminated samples. The contaminated mussel samples were removed from their shells, homogenized and the homogenate stored frozen at −20 °C until required. Samples (1 g of homogenate) were weighed into centrifuge tubes and 4 mL of PBS buffer (pH 7.2) was added. Each sample was then extracted and the supernatant collected as described previously. Triplicate 25 mg batches of microspheres were incubated with 1 mL of naturally contaminated supernatant at room temperature with constant agitation for 10 minutes. The Ferrospheres-N were then washed three times with PBS to remove any unbound toxin. Bound toxin was eluted with the addition of 0.1 M glycine/HCl (pH 2.5) and agitation for 2 minutes. Samples from each step were analyzed by HPLC [7]. The toxin recovered in the elution step was then compared to toxin extracted via the acetic acid extraction procedure and SPE clean-up/fractionation reported in the AOAC HPLC method [7].

### 2.7. HPLC Procedure for PSP Toxins

PSP toxins were analyzed after pre-chromatographic oxidation by the method described in the AOAC Official method 2005.06 ([7] AOAC, 2006). Briefly, the PSP toxins are oxidised to fluorescent derivatives by peroxide and periodate oxidation prior to separation using a Supelcosil LC-18 reversed-phase column (15cm × 4.6 mm and 5 μm particle size). The HPLC-FD system was a Waters 2695 separations module, equipped with a mobile-phase degasser and a Waters multi λ fluorescence detector. Data were analyzed by the Empower 2 software. The column was eluted with a gradient program of 2 mobile phases (Mobile phase-(A) 0.1 M ammonium formate; (B) 0.1 M ammonium formate in 5% acetonitrile, both adjusted to pH 6 with 0.1 M acetic acid), as follows: 0-5% mobile phase B during the first 5 minutes, then 5-70% during the next 4 minutes, then 70-0% over the next 2 minutes, and finally, 0% for another 3 minutes. The flow rate was maintained at 2.0 mL/min. STX and NEO were detected with the above mentioned fluorescence detector with an excitation wavelength of 330 nm and an emission wavelength of 395 nm.

## 3. Results and Discussion

### 3.1. Ferrospheres-N

The study describes the preliminary investigation of the viability of utilizing magnetic Ferrospheres-N in the absorption of STX from solution, in particular low concentrations of toxin. The amine-functionalized hollow glass magnetic Ferrospheres-N are a new innovation and as such offer a novel method for toxin extraction, particularly from algal culture, but also as a method of toxin extraction/purification from other solutions when coupled to antibodies highly specific to individual toxins. 

#### 3.1.1. Determination of the Antibody Binding Capacity of the Ferrospheres-N

The protein bound per gram of the GT-13A coupled Ferrospheres-N was determined with the modified BCA assay (Pierce) with BSA as the standard. The mass of GT-13A, per gram of microspheres was estimated to be 5.8 ± 0.3 mg/g. This resulted in a slightly higher binding capacity than the estimated binding capacity of 3-5 mg/g of rabbit IgG as supplied by the manufacturer.

#### 3.1.2. Elution Volume Needed

**Figure 2:**
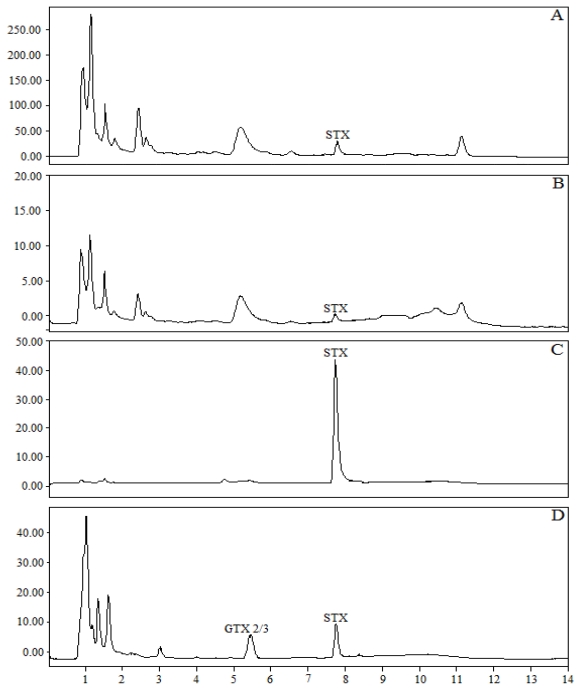
Influence of elution volume on recovery of STX from GT-13A Ferrospheres-N.

The GT-13A coupled Ferrospheres-N were subjected to an increasing volume of glycine/HCl elution buffer after the incubation of 100 ng/mL of STX in 1 mL for 2 minutes. Figure 2 shows that eluting the Ferrospheres-N with ≥1 mL of glycine/HCl resulted in a high recovery of bound STX (~86%). A one-way ANOVA indicated no statistical significance between any of the elution volumes used, F (4, 10) = 1.589 *p *= 0.26. As 1 mL of glycine/HCl was effective at eluting all bound STX it was used as the selected volume in all elution steps regarding the coupled Ferrospheres-N in this study.

#### 3.1.3. Reusability

To test the longevity of the bead performance, the STX binding-elution procedure was examined after each tenth use. The data shows the Ferrospheres-N allow for permitted repeated use, under the relatively mild elution conditions used in the study, and there was little marked reduction in toxin recovery until their thirty-fifth use when a decrease of ~8% was observed (Table 2). 

**Table 2:** Recovery of STX using Ferrospheres-N coupled to GT-13A after multiple uses.

Bead Usage	Mean (±SD) STX Eluted (ng)	% Recovery normalized against recovery obtained after 1^st^ usage
1	64.5 (±3.2)	100
10	64.0 (±7.6)	99.2
20	63.8 (±4.2)	98.9
30	62.9 (±5.3)	97.5
35	59.3 (± 11.1)	91.9

#### 3.1.4. Effect of STX concentration on the Recovery of STX from Spiked Buffers and Mussels

**Figure 3:**
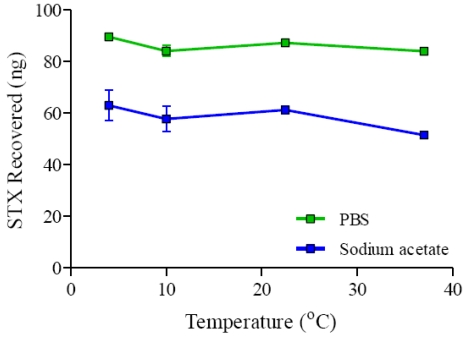
STX recovery from spiked buffers and spiked mussel extracts after to GT-13A coupled Ferrospheres-N.

To determine the efficiency of these novel spheres as means of capturing STX from spiked buffer and spiked mussel extracts, a range of spiked PBS and sodium acetate buffer and mussel extracts containing STX were prepared. Aliquots of the GT-13A Ferrospheres-N were added to these solutions to determine the recovery of STX. Figure 3 shows the curves obtained with the spiked samples. The Langmuir shape of the graphs for both the spiked sodium acetate buffer and spiked mussel extracts were expected as the toxin binding capacity of the Ferrospheres-N in sodium acetate and PBS buffer and spiked mussel extracts are reached. The graphs plateau and reach capacities of approximately 78 ng and 80 ng per 25 mg of microspheres for the spiked sodium acetate buffer and spiked mussel extracts respectively, and 183 ng and 142 ng per 25 mg of microspheres for spiked PBS and for spiked PBS mussel extracts respectively. STX recovery was also calculated for the effluent and all wash steps by comparison to a standard curve of STX standards in the respective solution (Data not shown). The majority of STX not accounted for in the elution step was accounted for in the effluent step with a negligible amount present in the wash step.

Although the trend observed in the curves is similar, the binding of STX from the sodium acetate mussel extract is slightly higher than the spiked sodium acetate buffer on its own. This slight enriching effect may be due to the mussel matrix raising or buffering the pH of the extract slightly from that of the sodium acetate buffer (pH 5.0). However, a lower amount of the STX was captured from the sodium acetate buffer and mussel extracts that the PBS buffer and mussel extracts over the similar range tested (0-300 ng/mL). The difference in the capture of STX between the two solutions may be due to the pH of the PBS buffer (7.2) being more optimal for STX binding to the GT-13A MAb than the sodium acetate buffers (pH 5.0).

From the graph the toxin recovery at 160 ng/mL of STX, which is equivalent to the regulatory limit of 800µg STX eq/kg shellfish, in spiked PBS mussel extract can be estimated to be in the region of 75%. This level of recovery is very comparable to the recovery of STX (74-93% at variable concentrations) from spiked mussel homogenates using the SPE sample preparation method in the AOAC 2005.06 method [7].

#### 3.1.5. Effect of Time on Recovery of STX from Spiked Mussel Extracts

**Figure 4:**
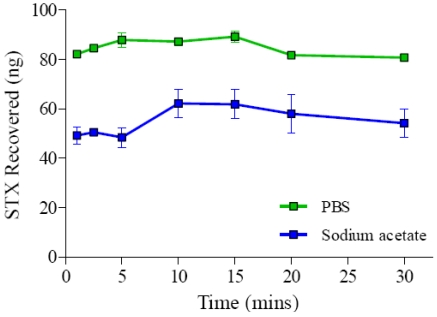
Effect of time on STX recovery from spiked mussel extracts.

To determine the effect of incubation time with the GT-13A coupled Ferrospheres-N, 100 ng/mL of STX in spiked sodium acetate or PBS mussel extracts, were added to the microspheres over a range of times (1-30 minutes). Figure 4 shows that the binding of STX remained relatively constant throughout the times tested. A one-way ANOVA was performed for STX recovery in spiked PBS mussel extract and found to be significant, F (6, 7) = 5.3, p = 0.02. However, Tukey post-hoc tests indicate that only 15 and 30 minutes of incubation differ significantly and the other groups were not statistically significant from each other (p = 0.05). The binding of STX remained relatively constant in the PBS between 5 and 15 minutes and thus 10 minutes was chosen for the incubation time throughout the study. The binding of STX remained relatively constant in the spiked sodium acetate mussel extracts over the time period tested (ANOVA, F (6, 7) = 1.26, *p* = 0.38). The short incubation time found to be required for spiked mussel extracts lends itself towards a highly rapid and easy to use sample preparation technique. The STX recovered was always found to be higher in the PBS spiked mussel than the sodium acetate spiked mussel. This may be due to the fact that PBS buffer (pH 7.2) is at a more favorable pH for antibody-antigen (toxin) interactions than the sodium acetate buffer (pH 5.0). The remaining STX not bound and eluted from the Ferrospheres-N in the elution step was mainly found in the effluent of the STX spiked sodium acetate and PBS extracts with a negligible quantity observed in the PBS wash step.

#### 3.1.6. Effect of Temperature on Recovery of STX from Spiked Mussel Extracts

**Figure 5:**
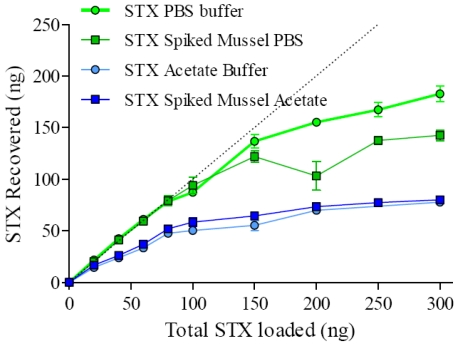
Effect of temperature on STX recovery from spiked mussel extracts.

Effect of temperature on the binding of STX onto the GT-13A immobilized Ferrospheres-N has been presented in Figure 5. Binding of STX on the Ferrospheres-N remains relatively constant at all temperatures tested in the extraction from spiked PBS mussel extract (ANOVA, F(3, 4) = 4.051, *p* = 0.105) and spiked sodium acetate mussel extract (ANOVA, F(3, 4) = 1.67, *p* = 0.31). The STX recovered from spiked PBS mussel extracts was again higher that the STX recovered from spiked sodium acetate mussel extracts. Again this is probably due to the more optimal pH of the PBS buffer for immuno-capture. The relatively constant binding of STX over the range of temperatures tested suggests the extraction of STX from sodium acetate and PBS mussel extracts can be performed with similar recoveries over the temperature range tested and may lead to their use in areas outside of the laboratory, such as field studies.

#### 3.1.7. PSP Toxin Extraction from Naturally Contaminated Mussel Samples

Table 3 shows the PSP toxins recovered from 1 mL of mussel supernatant after extraction with PBS using the GT-13A coupled Ferrospheres-N. When compared to the AOAC HPLC method [7] approximately 84% of STX but less than 1% of the NEO and GTX 1/4 were captured and eluted from the microspheres. The low recoveries of NEO and GTX 1/4 were not unexpected given the cross reactivity profile of GT-13A characterized by ELISA and surface plasmon resonance [16,17]. 

**Table 3:** PSP toxin recovery as determined by the AOAC HPLC method, after addition and elution from Ferrospheres-N and percentage recovery of the microsphere method when compared to the HPLC extraction method.

Sample	Ronas Voe			Cribba Sound		
Toxin	AOAC HPLC method (ng/mL)	Recovered after Ferrospheres-N (ng/mL)	% Recovery	AOAC HPLC method (ng/mL)	Recovered after Ferrospheres-N (ng/mL)	% Recovery
STX	83	69.6 ± 4.1	83.9 ± 4.9	179	67.6 ± 3.3	39.3 ± 1.9
NEO	ND	ND	ND	112	ND	ND
GTX 1/4	437	4.2 ± 2.5	1.0 ± 0.6	417	3.8 ± 0.4	0.9 ± 0.1
GTX 2/3	302	188.1 ± 6.7	62.3 ± 2.2	268	122.8 ± 34.9	45.8 ± 13.0
GTX 5	ND	1.6 ± 0.6		ND	3.3 ± 0.1	
C1/C2	ND	22.2 ± 5.6		ND	20.2 ± 1.4	

Another limiting factor is the toxin binding capacity of the microspheres used throughout this study. However, only 25 mg batches of GT-13A coupled Ferrospheres-N were used in this study, therefore the recovery of STX could be increased proportionately if an increased amount of GT-13A coupled Ferrospheres-N were used. A previous report demonstrated that an increase in the mass of human serum albumin (HSA) bound magnetic microspheres increased the percentage of ochratoxin A trapped onto the microspheres until 100% of the toxin was bound [18]. The antibody used in the study (GT-13A) is not suitable for the immune-capture of the R1-hydroxylated toxins however a cocktail of antibodies with different cross reactivity profiles to the PSPs would prove very suitable. 

The analysis of the elution step also showed up one surprising aspect. Detectable levels of GTX 5 and C1/C2 toxins where recorded in the elution stage. This is surprising given that GTX 5 and C1/C2 toxins were not recorded after the standard AOAC HPLC extraction method. They may have been present at low levels and lost via the SPE clean-up/extraction steps of the HPLC method. This indicates that the Ferrospheres-N may have an enrichment effect on low levels of R1-non-hydroxylated toxins. 

**Figure 6:**
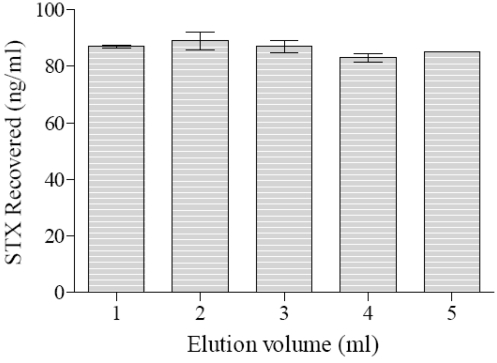
HPLC chromatograms showing the recovery of STX with GT-13A coupled Ferrospheres-N (a) effluent step, showing unbound toxin. (b) Wash step. (c) Elution step. (d) Comparison HPLC chromatogram of a mussel sample cleaned-up via the ‘Lawrence method’ [7].

This study on using immunoaffinity extraction of STX by antibody coated Ferrospheres-N has indicated that it may be a convenient alternative to conventional extraction procedures used in toxin purification. The Ferrospheres-N have a number of important advantages over column-based purification of toxins such as elimination of column fouling (due to matrix components). Additionally the Ferrospheres-N can be easily removed from the sample solution via the Magpen due to the Ferrospheres-N unique buoyancy property, *i.e.* they float on the surface of liquids due to the air filled cavity each microsphere possesses. This also permits the Ferrospheres-N to be thoroughly washed to remove any interfering matrix components prior to the specific election of the toxin and allows repetitive usage without any adverse effects on performance being noticed. The GT-13A coupled Ferrospheres-N also provided excellent chromatographic clean-up from the spiked mussel samples. Figure 6 shows that the majority of compounds causing non-toxin peaks after pre-chromatographic oxidation and HPLC do not bind to the microspheres (Figure 6) and that the remaining compounds are removed via washing (Figure 6b). The elution chromatogram (Figure 6c) contains no interfering peaks in contrast to the early eluting peaks, which interfere with early eluting toxins, in the naturally contaminated mussel sample (Figure 6d) after extraction by the method present in AOAC Official Method 2005.06 [7].

The antibody coated Ferrospheres-N may have an important application in the preparation of reference standards for PSP toxins, due to their specificity, ease of use and low cost, as PSP toxin purification is currently time consuming and difficult to achieve and requires the complicated and repetitive use of various forms of liquid chromatography [19,20,21]. The removal of toxins from algal culture or contaminated shellfish extracts are widely employed to prepare reference materials for PSP analysis and the microsphere approach may find a place in conjunction with other separation and purification methods in the preparation of individual toxin standards. The worldwide PSP problems requires a plentiful supply of highly purified PSP toxins for use in PSP monitoring laboratories and research institutes for the improvement, development and routine use of monitoring methods. 

To date no use of immuno-magnetic microsphere methods for PSP toxin extraction has been reported. However, immuno-magnetic bead-based methods have been developed within the area of food toxicology. Magnetic bead usage within the food toxicology field mainly concern magnetic bead-based immunoassays. Examples of such assays include a fluorescence-based immunoassay for staphylococcal enterotoxin B, such as the one reported by [22]; an immunomagnetic bead assay for botulinum neurotoxin types C and D [23]; an electrochemical based immunoassay for zearalenone reported by Hervás *et al.* [24]; an enzyme linked immuno-magnetic electrochemical (ELIME) methods for mycotoxin detection [25] and aflatoxin B-1 in corn samples [26]. Therefore, the use of magnetic microsphere immunoassays for the detection of PSP toxins may be a potential area for future research.

## 4. Conclusions

The preliminary investigation into the use of the MAb Ferrospheres-N showed that the GT-13A was successfully coupled to the microspheres and that the capture of spiked STX from solution was feasible. Further investigation also revealed that the binding of STX by the Ferrospheres-N is relatively constant over the incubation times and temperatures tested within this study and showed the potential speed and versatility of the method. 

Coated Ferrospheres-N microspheres showed potential in extracting PSP toxins from naturally contaminated mussel samples. Limitations of the cross-reactivity of the antibody and the mass of microspheres used throughout the study were apparent. Total PSP toxin recovery could potentially be increased by using a greater amount of GT-13A-coupled Ferrospheres-N and an antibody with an improved cross-reactivity for the complement of PSP toxins or a cocktail of antibodies. Therefore, this study demonstrates that the use of antibody-coupled Ferrospheres-N offers a potentially rapid extraction method for PSP toxins from contaminated samples for HPLC analysis or the preparation of reference quality materials depending on the antibody coupled to the microspheres.
